# Supplementary material for: Serial imaging of micro-agents and cancer cell spheroids in a microfluidic channel using multicolor fluorescence microscopy
Source: PLoS One. 2021 Jun 15;16(6):e0253222. doi: 10.1371/journal.pone.0253222 (PMC8205435; doi:10.1371/journal.pone.0253222)
Supplement: S1 Fig — Schematic representation of the demultiplexer interface used for preventing crosstalk between fluorophores by synchronically triggering light-emitting diodes (LEDs) and complementary metal-oxide-semiconductor (CMOS) cameras in a round-robin manner using two individual pulse trains. (a and c) Architecture and computed frequency response plot of the demultiplexer circuit. (b) Timing diagram for input pulse trains generated using a signal generator (33510B, Keysight, USA) and output trigger signals for each LED and CMOS sensor combination. (PDF) [file pone.0253222.s001.pdf]

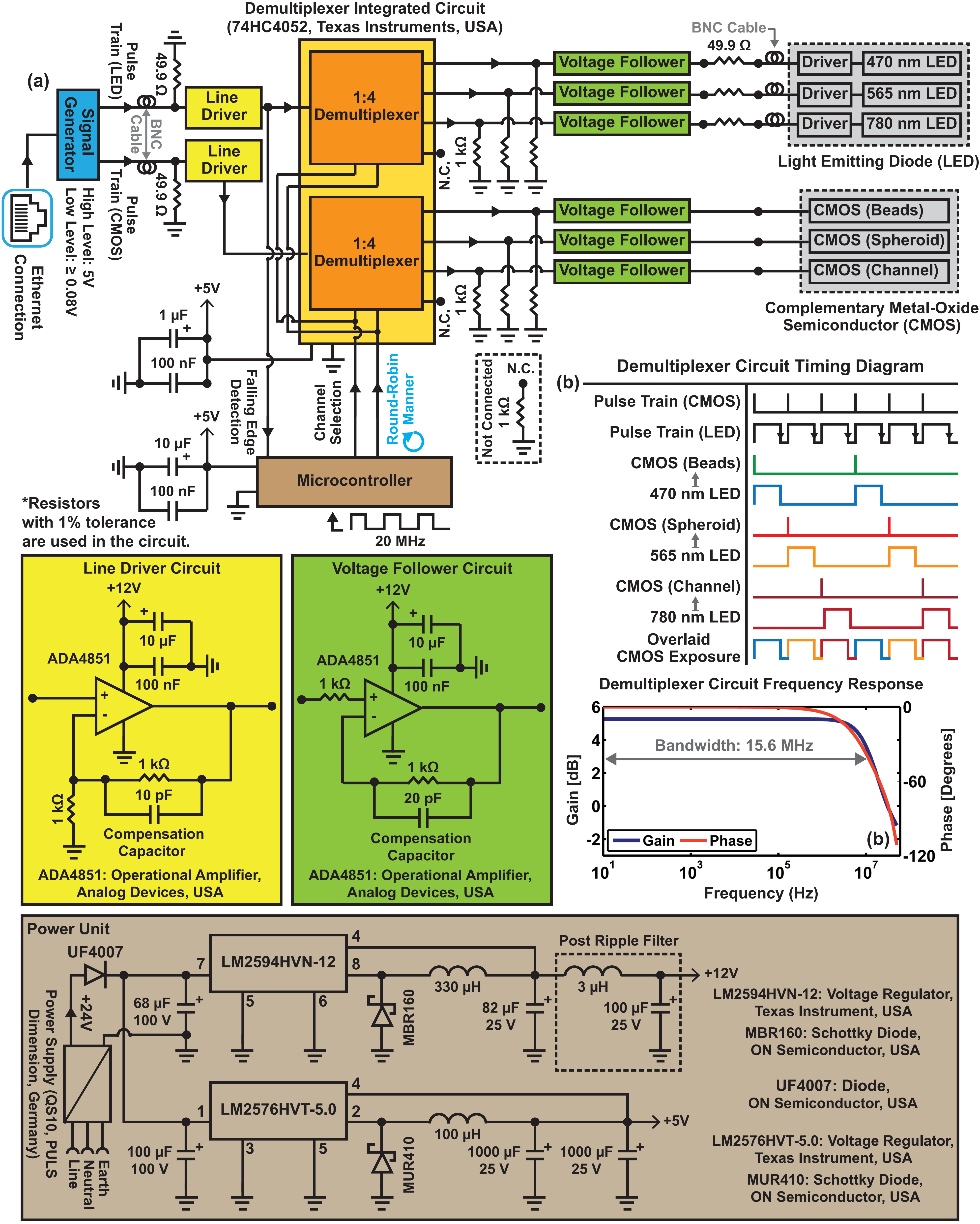
(a)
Demultiplexer Integrated Circuit
(74HC4052, Texas Instruments, USA)
Signal Generator
Ethernet Connection
High Level: 5V
Low Level: ≥ 0.08V
Pulse Train (LED)
Pulse Train (CMOS)
BNC Cable
49.9 Ω
Line Driver
Line Driver
1:4 Demultiplexer
1:4 Demultiplexer
N.C.
1 kΩ
Voltage Follower
BNC Cable
49.9 Ω
Driver
470 nm LED
565 nm LED
780 nm LED
Light Emitting Diode (LED)
CMOS (Beads)
CMOS (Spheroid)
CMOS (Channel)
Complementary Metal-Oxide Semiconductor (CMOS)
+5V
1 µF
100 nF
10 µF
Falling Edge Detection
Channel Selection
Round-Robin Manner
Not Connected
1 kΩ
Microcontroller
20 MHz
*Resistors with 1% tolerance are used in the circuit.
(b)
Demultiplexer Circuit Timing Diagram
Pulse Train (CMOS)
Pulse Train (LED)
CMOS (Beads)
470 nm LED
CMOS (Spheroid)
565 nm LED
CMOS (Channel)
780 nm LED
Overlaid CMOS Exposure
Line Driver Circuit
+12V
10 µF
100 nF
ADA4851
1 kΩ
10 pF
Compensation Capacitor
ADA4851: Operational Amplifier, Analog Devices, USA
Voltage Follower Circuit
+12V
10 µF
100 nF
ADA4851
1 kΩ
20 pF
Compensation Capacitor
ADA4851: Operational Amplifier, Analog Devices, USA
Demultiplexer Circuit Frequency Response
Bandwidth: 15.6 MHz
Gain [dB]
Phase [Degrees]
Gain
Phase
(b)
6
4
2
0
-2
0
-60
-120
10^1
10^3
10^5
10^7
Frequency (Hz)
Power Unit
UF4007
+24V
Power Supply (QS10, PULS Dimension, Germany)
Line
Neutral
Earth
68 µF 100 V
LM2594HVN-12
7
8
4
5
6
MBR160
330 µH
82 µF 25 V
Post Ripple Filter
3 µH
100 µF 25 V
+12V
LM2594HVN-12: Voltage Regulator, Texas Instrument, USA
MBR160: Schottky Diode, ON Semiconductor, USA
UF4007: Diode, ON Semiconductor, USA
LM2576HVT-5.0
1
2
3
4
5
100 µF 100 V
MUR410
100 µH
1000 µF 25 V
1000 µF 25 V
+5V
LM2576HVT-5.0: Voltage Regulator, Texas Instrument, USA
MUR410: Schottky Diode, ON Semiconductor, USA
