# Supplementary material for: Serial imaging of micro-agents and cancer cell spheroids in a microfluidic channel using multicolor fluorescence microscopy
Source: PLoS One. 2021 Jun 15;16(6):e0253222. doi: 10.1371/journal.pone.0253222 (PMC8205435; doi:10.1371/journal.pone.0253222)
Supplement: Attachment — Submitted filename: Rebuttal(15.05.2021).pdf [file pone.0253222.s002.pdf]

Paper Title: Serial imaging of micro-agents and cancer cell spheroids in a microfluidic channel using multicolor fluorescence microscopy

Paper ID: PONE-D-21-10897

Dear Professor Maitland,

In response to your email (dated 29 April 2021), we would like to thank you and the reviewers for the detailed comments. We have revised the letter accordingly, and the changes made are highlighted in red for clarity. We would like to thank the anonymous reviewers for their constructive comments.

Sincerely,

Mert Kaya
Doctoral Candidate
Surgical Robotics Laboratory
Department of Biomechanical Engineering
University of Twente, The Netherlands
E-mail: m.kaya@utwente.nl
Phone: +31-(0)53-489-1449
Website: www.surgicalroboticslab.nl

Reviewer #1: In this manuscript the authors describe and evaluate a fluorescence microscope custom designed for efficient three-channel fluorescence imaging. The microscope design is thoroughly explained and presented by a diagram (Fig. 1). The custom multi-color microscope is demonstrated for imaging microparticles and cancer spheroids within channels and the results of characterization experiments for photobleaching, aberration artifacts, and spectral overlap are presented and discussed. The performance of the multi-color microscope is compared with a brightfield microscope and a traditional fluorescence microscope. Both qualitative and quantitative results are included.

Response: Thank you for your kind words.

Reviewer #2: Kaya *et al.* presented a widefield microscopy scheme capable of imaging 3 fluorophores with distinct emission profiles. The developed system was demonstrated by imaging labeled tumor spheroid and fluorescent beads loaded in a microfluidic channel stained with a third fluorophore. My main concern is that the novelty of the manuscript is not convincing. The authors applied an existing microscopy technique to new samples, however no new biological findings are reported. Multicolor fluorescence imaging is very common and is extensively used in research labs.

We would like to thank you for your constructive comments. You can find our responses to your comments below:

R2.1. Introduction, page 1, line 6-8. The authors state the advantages of using fluorescence microscopy. However the reasons stated can be true for any light microscopy technique and does not seem specific for fluorescence microscopy.

Response: The sentence was revised as:

> Line 6-9: "Among the microscopy techniques, fluorescence microscopy is the most effective way to visualize a specific target structure with a relatively high contrast-to-noise ratio by eliminating the background."

R2.2. Introduction section, page 2, line 19-20, the authors mention that fluorescence microscopy is used for samples that are stained. This is not true as endogenous fluorophores or fluorescent proteins can imaged using this technique too. Also, rest of the paragraph consists cursory definition of fluorescence an its properties, which seems more appropriate for a tutorial style paper. Moreover, no references have been included.

Response: The definition of fluorescence imaging was removed from the text. The sentence (page 2, line 19-20) was revised and relevant references were added:

Line 19-21: "The working principle of this microscopy technique is based on performing imaging using spectrally-different conventional fluorescent dyes [11], endogenous fluorophores [22], fluorescent proteins [24], or quantum dots [25]. "

[11] Park KS, Kim DU, Lee J, Kim GH, Chang KS. Simultaneous multicolor imaging of wide-field epi-fluorescence microscopy with four-bucket detection. Biomedicaloptics express. 2016 Jun 1; 7(6):2285-94.

[22] Stringari C, Abdeladim L, Malkinson G, Mahou P, Solinas X, Lamarre I, BrizionS, Galey JB, Supatto W, Legouis R, Pena AM. Multicolor two-photon imaging ofendogenous fluorophores in living tissues by wavelength mixing. Scientific reports.2017 Jun 19; 7(1):1-1

[24] Hu C, Kerppola T. Simultaneous visualization of multiple protein interactions inliving cells using multicolor fluorescence complementation analysis. NatureBiotechnology. 2003;21(5):539-545.

[25] Larson DR, Zipfel WR, Williams RM, Clark SW, Bruchez MP, Wise FW, WebbWW. Water-soluble quantum dots for multiphoton fluorescence imaging in vivo.Science. 2003; 300(5624):1434-1436.

R.2.3. HeLa cell spheroid with culture media does not "create a tumor micro-environment" which would involve other cells like immune cells, cancer associated fibroblast, extracellular matrix , nutrient, oxygen and pH gradients and so on. The authors develop a simplified 3D tumor model. Please consider re-phrasing.

Response: The sentences were revised as:

Abstract: "To report the performance of the multicolor microscopy, a simplified 3D tumor model is created by placing beads and spheroids inside a channel filled with the cell culture medium is imaged at varying exposure times."

Line 42-45: "For imaging experiments, cancer cell spheroids with a diameter of 200 μm are formed using HeLa cells and placed inside a microfluidic channel filled with cell culture medium to create a simplified 3D tumor model."

Line 172-173: "To create a simplified 3D tumor model, HeLa cell spheroids stained with CMTPX are embedded in microfluidic channels with a height of 187 μm using a micropipette."

> Line 421-423: "We develop a simplified 3D tumor model to validate spectrally-different image acquisition of beads, spheroids, and channel, using multicolor fluorescence microscopy."

R.2.4. Please justify the diameters chosen for Hela cell spheroid and polystyrene beads. Are these clinically relevant values?

Response: We chose the diameter of the polystyrene beads as surrogates for drug carriers and HeLa cell spheroid on the clinical study in [1] and microfluidic channel-based drug delivery experiments performed in [2].

> **[1]** Pouponneau P, Bringout G, Martel S. Therapeutic magnetic microcarriers guided by magnetic resonance navigation for enhanced liver chemoembilization: a design review. Annals of biomedical engineering. 2014 May; 42(5):929-939.
>
> **[2]** Xu H, Medina-Sánchez M, Magdanz V, Schwarz L, Hebenstreit F, Schmidt O. Sperm-Hybrid Micromotor for Targeted Drug Delivery. ACS Nano. 2017; 12(1):327-337.

The diameter of the micro-agents used for liver chemoembolization in [1] is in the range of 16 µm to 65 µm. The sperms with a head diameter of 10 µm were functionalized as drug carriers in [2]. They were moved to the cancer cell spheroids with a diameter of 200 µm.

The following references were added to the manuscript:

> Line 42-45: "For imaging experiments, cancer cell spheroids with a diameter of 200 µm are formed using HeLa cells and placed inside a microfluidic channel filled with cell culture medium to create a simplified 3D tumor model. Polystyrene beads with a diameter of 20 µm are employed as surrogates for drug carriers and injected in the channel **[28-30].**"
>
> **[28]** Pouponneau P, Bringout G, Martel S. Therapeutic magnetic microcarriers guided by magnetic resonance navigation for enhanced liver chemoembilization: a design review. Annals of biomedical engineering. 2014 May; 42(5):929-939.
>
> **[29]** Xu H, Medina-Sánchez M, Magdanz V, Schwarz L, Hebenstreit F, Schmidt O. Sperm-Hybrid Micromotor for Targeted Drug Delivery. ACS Nano. 2017; 12(1):327-337.
>
> **[30]** Nelson BJ, Kaliakatsos IK, Abbott JJ. Microrobots for minimally invasive medicine. Annual review of biomedical engineering. 2010; 12(1):55-85.

R.2.5. Why was the widefield microscope built with capability of imaging those 3 specific fluorophores? Development an adaptable system that could be used for multiple application would be more useful. Please justify.

Response: Direct segmentation of micro-agents, spheroids, and microfluidic channel in the acquired individual fluorescence images is required for clear visualization. Fluorescein, CMPTX, and indocyanine green are well-established dyes, with fairly well-separated emission spectra and good stability against photobleaching. We chose these fluorophores to acquire crosstalk-free individual images using the round-robin method. Further, HeLa cell spheroids are stained with both CMPTX and indocyanine green to visualize their complete structure (Fig. 1). Thus, four-color fluorescence microscopy is performed using these three fluorophores.

Since our samples were defined in advance based on the studies in the literature and the same samples would be used in our future studies, we have never considered an adaptable system ([1-2]). We note that the standard optical components were especially used to visualize micro-agents from the green fluorescence band. Micro-agents are fabricated using polymers [3-4]. Different types of fluorophores like Coumarin 6 and Dyomics DY-478 can be used to stain the polymers used for fabrication of the micro-agents, which enables their visualization using the widefield microscope presented in the manuscript.

The following sentences were added to the manuscript:

> Line 342-344: “Therefore, four-color fluorescence microscopy is performed utilizing fluorescein, CMTPX, and indocyanine green for dynamic imaging of micro-agents and 3D cancer cell spheroids in a microfluidic setup.”
>
> Line 425-427: “To correct the spectral crosstalk, fluorophores with fairly well-separated emission spectra are excited in a round-robin manner and the emitted light is synchronously collected.”

[1] Ahn J, Ko J, Lee S, Yu J, Kim Y, Jeon NL. Microfluidics in nanoparticle drugdelivery; From synthesis to pre-clinical screening. Advanced drug delivery reviews.2018 Mar 15; 128:29-53.

[2] Xu H, Medina-Sánchez M, Magdanz V, Schwarz L, Hebenstreit F, Schmidt O. Sperm-Hybrid Micromotor for Targeted Drug Delivery. ACS Nano. 2017; 12(1):327-337.

[3] Mohanty S, Siciliani de Cumis U, Solsona M, Misra S. Bi-directional transportation of micro-agents induced by symmetry-broken acoustic streaming. AIP Advances. 2019;9(3):035352.

[4] Khalil, I. S., Magdanz, V., Sanchez, S., Schmidt, O. G., & Misra, S. (2013). Three-dimensional closed-loop control of self-propelled microjets. Applied physics letters, 103(17), 172404.

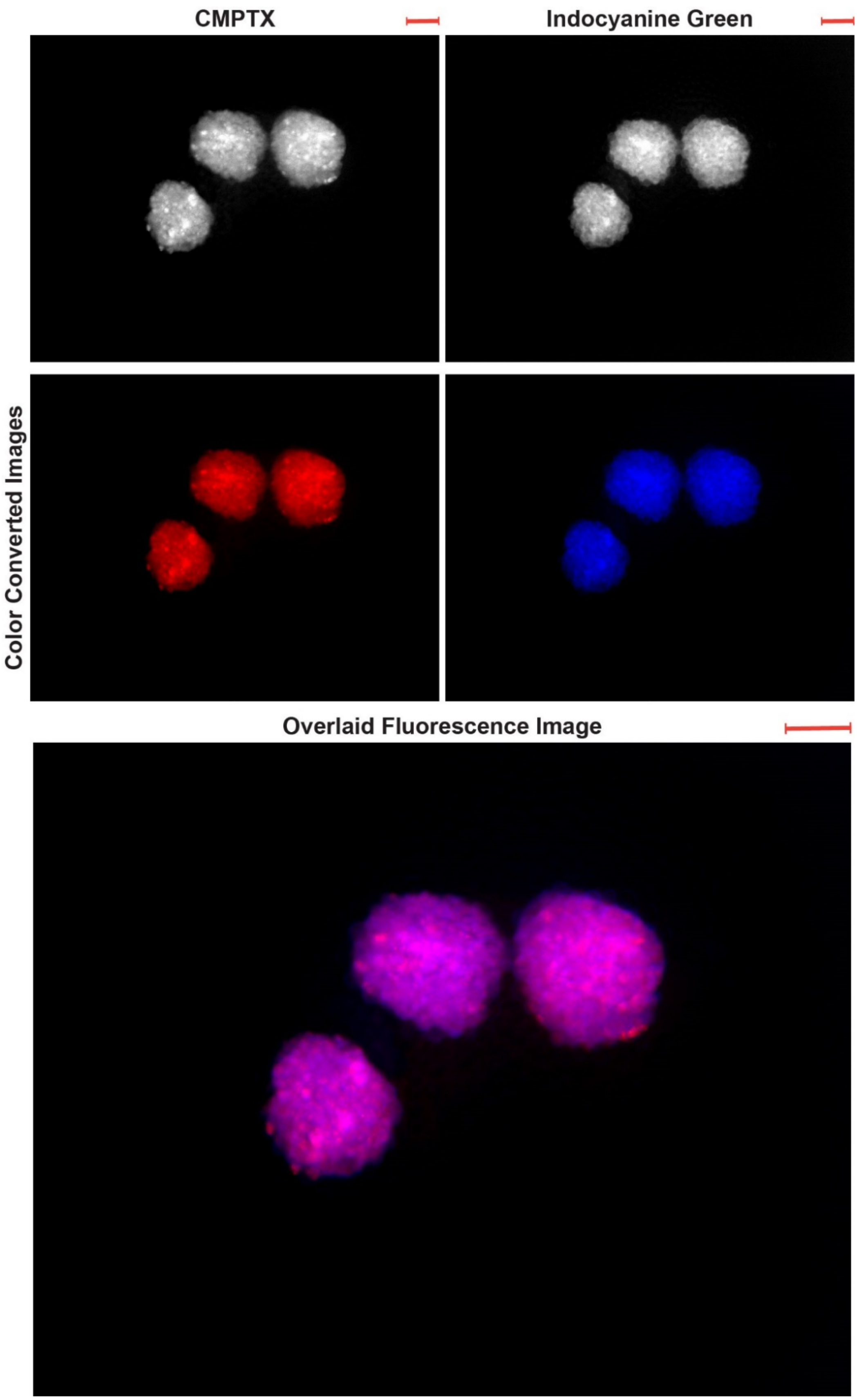

Fig. 1: Dual-Color fluorescence imaging of HeLa cell spheroids using CMPTX and indocyanine green. Scale bar: 100 µm.

R.2.6. Maybe I missed it but why was the microfluidic channel loaded with fixed HeLa cell spheroids instead of live cells, since fixed cells has no physiological relevance?

Response: HeLa cell spheroids were fixated for long-term storage. The following sentence was added to the manuscript.

> Line 167-169: "For long-term storage, HeLa cell spheroids stained with CMTPX are fixated for 15 minutes with 4\% formaldehyde (F8775-25ML, Sigma-Aldrich, USA) at room temperature."

R.2.7. From figure 4(d1) it seems like there is bleed through of CellTracker Red CMTPX in the indocyanine green channel.

Response: To study the bleeding, HeLa cell spheroids stained with CMPTX are placed inside the channel filled with Dulbecco's phosphate-buffered saline. Fluorescence images are acquired from 500–540 nm (fluorescein), 592.5–667.5 nm (CMPTX), and 817.5–875.5 nm (indocyanine green) spectrum bands using the round-robin method. Although images are acquired from the indocyanine green channel at maximum camera gain and excitation power, we did not observe the bleeding effect (Fig. 2). As we showed in the spectrum analysis (Fig 1. (a) and Fig 4. (a) in the manuscript), HeLa cells stained with CMPTX are not able to be imaged in the indocyanine green channel.

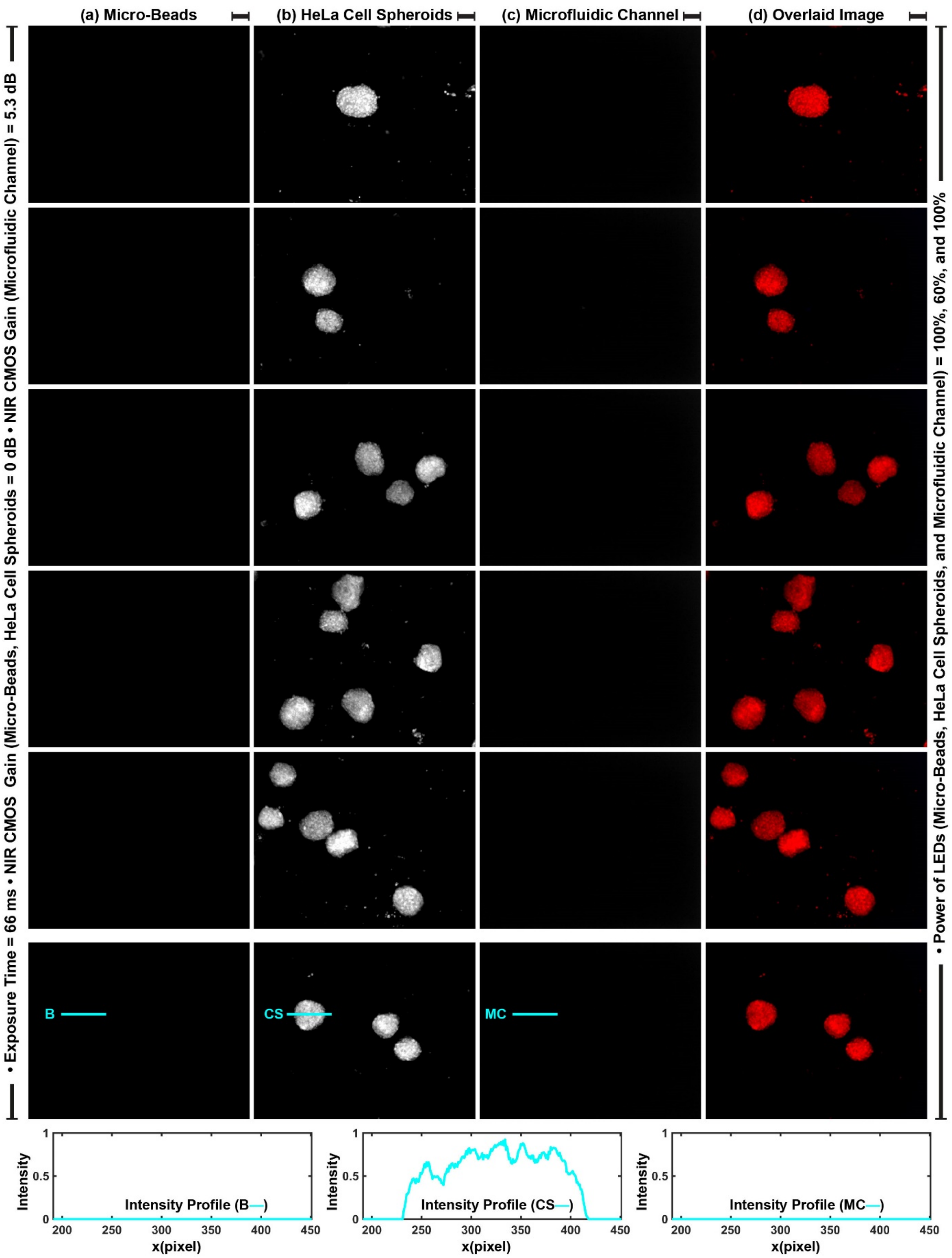

Fig. 2: Fluorescence imaging of HeLa cell spheroids stained with CMPTX. (a)-(c) Acquired fluorescence images from 500–540 nm, 592.5–667.5 nm, and 817.5–875.5 nm spectrum bands, respectively. (d) Formed image by overlapping acquired images. Scale bar: 100 µm.

R.2.8. Quantification of the data for cross-talk analysis would be more convincing along with the representative images shown.

Response: Structural similarity index measure metric is used for quantitative measurement of the crosstalk analysis:

> Line 279-296: "The crosstalk is corrected and the blockage is removed by exciting the fluorophores in a round-robin manner and synchronically trigger cameras for individual image acquisition (Fig. 6-(a8)). The effect of crosstalk on the individual images acquired using continuous (Fig. 6-(a7)) and round-robin (Fig. 6-(a8)) methods is quantified by image quality assessment using structural similarity index measure (SSIM) metric [41]. Single-band fluorescence images of micro-beads (500–540 nm band), HeLa cell spheroid (592.5–667.5 nm band), and microfluidic channel (817.5–875.5 nm band) are used as references for the measurements (Fig. 6-(a1)-(a3)). SSIM values for the individual images of beads (Fig. 6-(f1)) and spheroid (Fig. 6-(f2)) acquired using the continuous method are computed as 0.24 due to the crosstalk and the blockage, respectively. Individual images of the channel are acquired using both continuous and round-robin methods with the SSIM value of about 0.91 (Fig. 6-f(3) and g(3)). Since indocyanine green is not able to be excited with 470 nm and 565 nm LEDs and the emission filter (ET845/55m, Chroma, USA) blocks the fluorescent photons emitted from fluorescein and CMTPX (Fig. 1). We compute that the individual images are acquired using the round-robin method with the SSIM values of above 0.90, which enables spectral unmixing of micro-beads, HeLa cell spheroid, and microfluidic channel for dynamic real-time imaging (Fig. 6-(g))."

[41] Wang Z, Bovik AC, Sheikh HR, Simoncelli EP. Image quality assessment: from error visibility to structural similarity. IEEE transactions on image processing. 2004 Apr 13; 13(4):600-12.

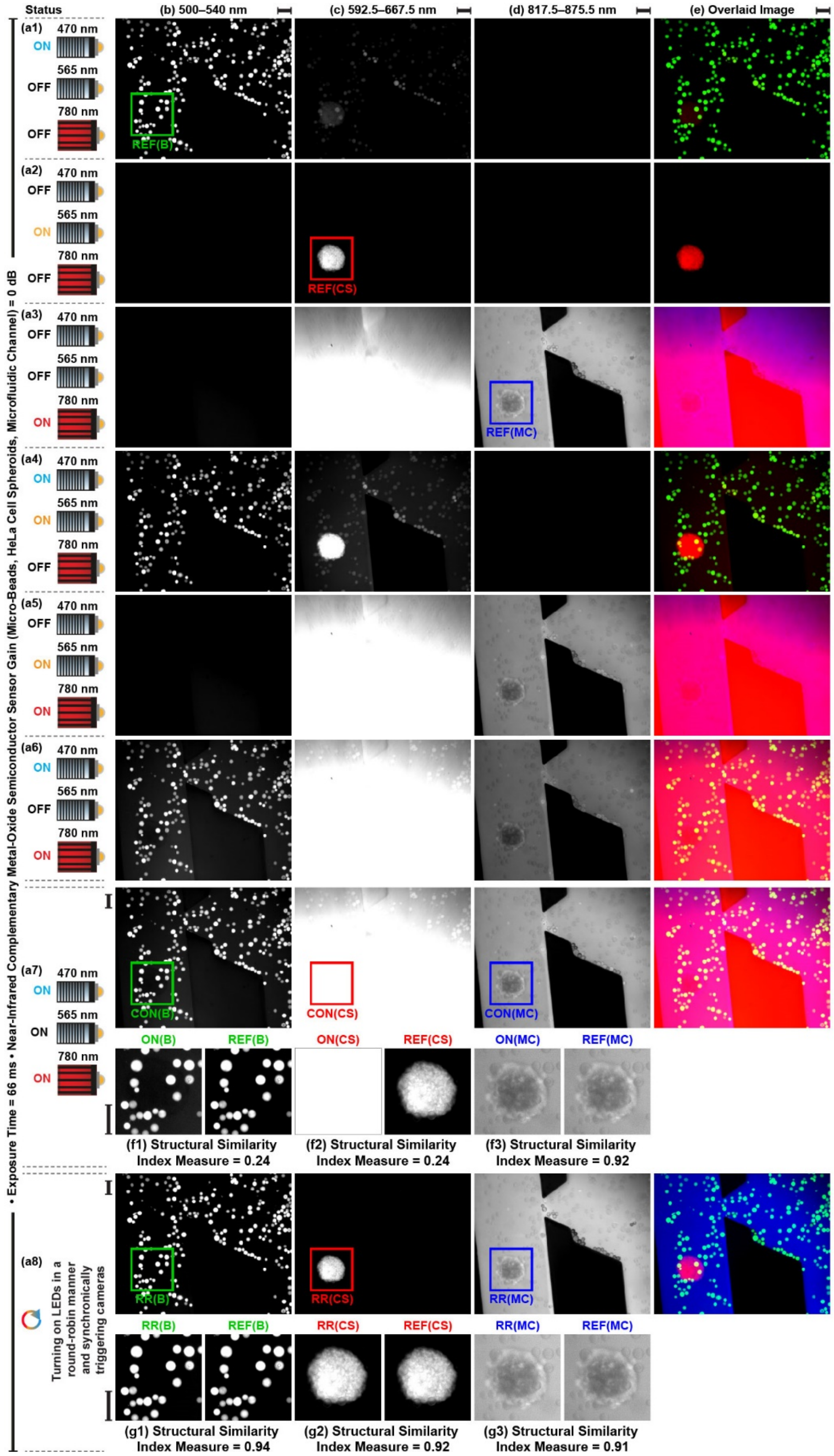

Fig 6. Crosstalk analysis. (a) Status of light-emitting diodes. (b)-(d) Acquired fluorescence images from 500-540 nm, 592.5-667.5 nm, and 817.5-875.5 nm spectrum bands, respectively. (e) Formed image by overlapping acquired images. (f) and (g) Quantitative crosstalk analysis by computing structural similarity measure index between reference images (REF) and images acquired using continuous (CON) and round-robin (RR) methods, respectively. Scale bar: 100 µm.

R.2.9. The electrostatic interaction between beads and HeLa spheroids is confusing and does not provide additional data. The data shown are just images showing location of bead attachments on the spheroids. This does not necessarily provide information about the interaction.

Response: Imaging of electrostatic interaction between polystyrene micro-beads and HeLa cell spheroid section and the corresponding figure were polished as (line: 399-416) :

"Real-time multicolor fluorescence microscopy

HeLa cell spheroids and polystyrene micro-beads are negatively and positively charged, respectively. Utilizing electrostatic forces, beads are able to be attached to the surface of spheroids without functionalization, which a self-assembly method used for bio-hybrid micro-agent fabrication [46]. As a representative application, the attachment of micro-beads to a HeLa cell spheroid in a microfluidic channel is imaged using multicolor fluorescence microscopy. For the imaging experiment, the channel is fixed on the sample holder attached to a translation stage (PT3/M, Thorlabs, USA). The holder is manually and randomly moved in horizontal and vertical directions until no further bead capture by the spheroid takes place due to the neutralization (Fig. 11). Individual fluorescence images of the beads, spheroid, and channel are acquired with 22 ms exposure time and 5.3 dB gain at 15 fps and overlaid to form multicolor images. The motion of beads, spheroid, and channel between consecutive individual fluorescence images are analyzed using the Lukas-Kanade optical flow technique [47]. The analyzed motion at 63 seconds and 518 seconds is represented as three independent vector fields and overlaid onto the fluorescence images (Fig. 11-(c)). Our experiment shows that individual motion of micro-agents, 3D cancer cell spheroids, and microfluidic setup is able to be imaged in real-time by spectrally different image acquisition."

[46] Magdanz V, Khalil IS, Simmchen J, Furtado GP, Mohanty S, Gebauer J, Xu Haifeng, Klingner A, Aziz A, Medina-Sánchez M, Schmidt OG, Misra S. IRONSperm: Sperm-templated soft magnetic microrobots. Science advances. 2020 Jul 1; 6(28):eaba5855.

[47] Lucas BD, Kanade T. An iterative image registration technique with an application to stereo vision.

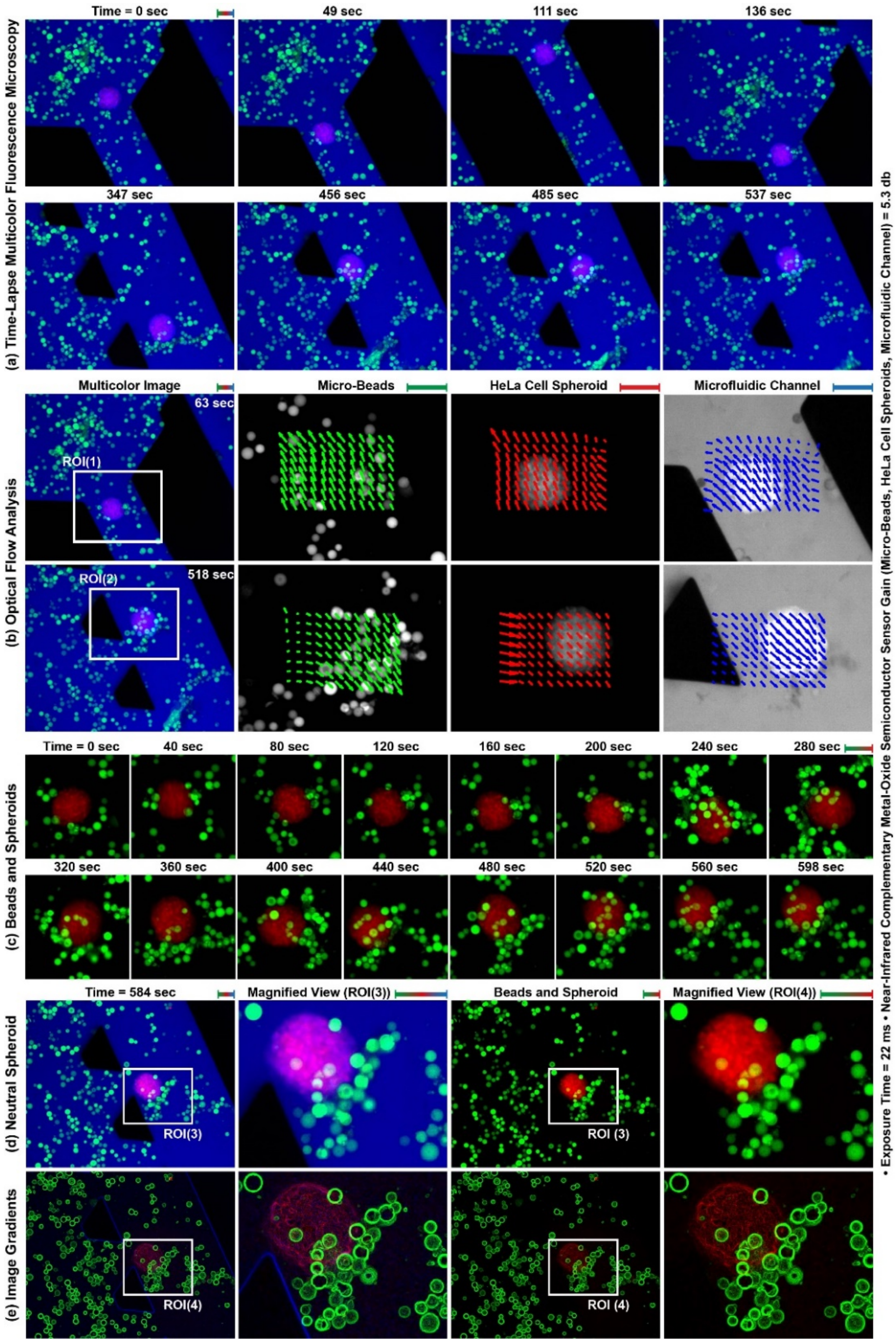

Fig 11. Multicolor fluorescence microscopy for the attachment of polystyrene micro-beads to a HeLa cell spheroid surface utilizing electrostatic forces. (a) Time-lapse multicolor fluorescence image sequence acquired at 15 frames per second. (b) The analyzed motion using Lukas-Kanade optical flow in ROI(1) and ROI(2) at 63 seconds and 518 seconds is represented as vector fields and overlaid on the fluorescence images. The spectrally different motion information of beads, spheroid, and channel is represented using green, red, and blue arrows, respectively. (c) Time-lapse fluorescence image sequence of beads and spheroid. (d) Neutral HeLa cell spheroid covered with micro-beads. (e) Gradients of the images in (d) for contrast visualization. Scale bar: 100 μm.

R.2.10. Advantages over previously published multicolor widefield systems (eg: https://doi.org/10.1364/BOE.7.002285) should be discussed along with disadvantage or shortcomings of the presented technique. Also discuss the advantages of using a 'round-robin' detection scheme

Response:

Advantage of the multicolor widefield microscope presented in the paper:

> Line 46-53: "In our simplified tumor model, there are height differences between beads, spheroids, and channel. To form focused multicolor fluorescence images using widefield microscopy, individual image acquisition from different focal planes is required. Since micro-structures in the previously used samples have almost the same height, defocus aberration correction is not addressed for widefield multicolor imaging [11, 18, 31, 32]. In this study, our multicolor fluorescence microscope is specially developed for defocus aberration correction by simultaneous image acquisition from multiple focal planes within the simplified tumor model."

Disadvantage of the multicolor widefield microscope presented in the paper:

> Line 300-303: "For simultaneous multicolor imaging, the maximum frame acquisition rate equals 1/(τ) [11, 13, 18]. Since a multicolor image is formed by the sequential acquisition of emitted fluorescence light from three different spectrum bands using the round-robin method, the maximum frame rate is computed as 1/(3τ)."

Advantages of using a 'round-robin' detection scheme:

> Line 279-296: "The crosstalk is corrected and the blockage is removed by exciting the fluorophores in a round-robin manner and synchronically trigger cameras for individual image acquisition (Fig. 6-(a8)). The effect of crosstalk on the individual images acquired using continuous (Fig. 6-(a7)) and round-robin (Fig. 6-(a8)) methods is quantified by image quality assessment using structural similarity index measure (SSIM) metric [39]. Single-band fluorescence images of micro-beads (500–540 nm band), HeLa cell spheroid (592.5–667.5 nm band), and microfluidic channel (817.5–875.5 nm band) are used as references for the measurements (Fig. 6-(a1)-(a3)). SSIM values for the individual images of beads (Fig. 6-(f1)) and spheroid (Fig. 6-(f2)) acquired using the continuous method are computed as 0.24 due to the crosstalk and the blockage, respectively. Individual images of the channel are acquired using both continuous and round-robin methods with the SSIM value of about 0.91 (Fig. 6-f(3) and g(3)). Since indocyanine green is not able to be excited with 470 nm and 565 nm LEDs and the emission filter (ET845/55m, Chroma, USA) blocks the fluorescent photons emitted from fluorescein and CMTPX (Fig. 1). We compute that the individual images are acquired using the round-robin method with the SSIM values of above 0.90, which

enables spectral unmixing of micro-beads, HeLa cell spheroid, and microfluidic channel for dynamic real-time imaging (Fig. 6-(g)).

Line 430-432: “Our measurements show that multicolor fluorescence microscopy using round-robin method provide clear visualization by containing direct segmentation of beads, spheroids, and channel.”

[11] Park KS, Kim DU, Lee J, Kim GH, Chang KS. Simultaneous multicolor imaging of wide-field epi-fluorescence microscopy with four-bucket detection. Biomedical optics express. 2016 Jun 1; 7(6):2285-94.

[13] Wang K, Liu TM, Wu J, Horton NG, Lin CP, Xu C. Three-color femtosecond source for simultaneous excitation of three fluorescent proteins in two-photon fluorescence microscopy. Biomedical optics express. 2012 Sep 1; 3(9):1972-7.

[18] Li Q, Chen P, Fan Y, Wang X, Xu K, Li L, Tang B. Multicolor fluorescence detection-based microfluidic device for single-cell metabolomics: simultaneous quantitation of multiple small molecules in primary liver cells. Analytical chemistry. 2016 Sep 6; 88(17):8610-6.

[31] Yang C, Hou V, Nelson L, Seibel E. Mitigating fluorescence spectral overlap in wide-field endoscopic imaging. Journal of Biomedical Optics. 2013;18(8):086012.

[32] Zimmermann T, Rietdorf J, Pepperkok R. Spectral imaging and its applications in live cell microscopy. FEBS Letters. 2003;546(1):87-92.

[39] Bates M, Dempsey GT, Chen KH, Zhuang X. Multicolor super-resolution fluorescence imaging via multi-parameter fluorophore detection. ChemPhysChem. 2012 Jan 16; 13(1):99.
